# Groupies in multitype random graphs

**DOI:** 10.1186/s40064-016-2705-4

**Published:** 2016-07-07

**Authors:** Yilun Shang

**Affiliations:** Department of Mathematics, Tongji University, Shanghai, 200092 China

**Keywords:** Random graph, Degree, Groupie, Multitype, 05C07, 05C80

## Abstract

A groupie in a graph is a vertex whose degree is not less than the average degree of its neighbors. Under some mild conditions, we show that the proportion of groupies is very close to 1/2 in multitype random graphs (such as stochastic block models), which include Erdős-Rényi random graphs, random bipartite, and multipartite graphs as special examples. Numerical examples are provided to illustrate the theoretical results.

## Background

A vertex in a graph *G* is said to be a *groupie* if its degree is not less than the average degree of its neighbors. Various properties of groupies have been investigated in deterministic graph theory (Ajtai et al. 1980; Bertram et al. 1994; Ho 2007; Mackey 1996; Poljak et al. 1995). For example, it was proved in Mackey (1996) that there are at least two groupies in any simple graphs with at least two vertices. Groupies were even found to be related to Ramsey numbers (Ajtai et al. 1980). More recently, Fernandez de la Vega and Tuza (2009) showed that, in Erdős-Rényi random graphs *G*(*n*, *p*), the proportion of vertices that are groupies is almost always very near to 1/2 as $n\rightarrow \infty$. Later the author Shang (2010) obtained a result of similar flavor in random bipartite graphs $G(n_1,n_2,p)$. It was shown that the proportion of groupies in each partite set is almost always very close to 1/2 if $G(n_1,n_2,p)$ is balanced, namely, $n_1=n_2$.

In this paper, we consider groupies in a more general random graph model, which we call *multitype random graphs*. Let *q* be a positive integer. Denote $[q]:=\{1,\ldots ,q\}$. Define the ‘gene’ for a multitype random graph as a weighted complete graph $K_q$ (having a loop at each vertex) on the vertex set [*q*], with a weight $\alpha _i>0$ associated to each vertex, and a weight $0\le \beta _{ij}\le 1$ associate to each edge *ij*. Note that $\beta _{ij}=\beta _{ji}$ since we deal with undirected graphs. We assume $\sum _{i=1}^q\alpha _i=1$. The multitype random graph $G(n,K_q)$ with gene $K_q$ is generated as follows. Let *n* be much larger than *q*, and let [*n*] be its vertex set. We partition [*n*] into *q* sets $V_1,\ldots ,V_q$ by putting vertex *v* in $V_i$ with probability $\alpha _i$ independently. Each pair of vertices $v\in V_i$ and $u\in V_j$ are connected with probability $\beta _{ij}$ independently (all the decisions on vertices and edges are made independently).

For $i=1,\ldots ,q$, let $N_i$ represent the number of the groupies in $V_i$. Thus, $N:=\sum _{i=1}^qN_i$ is the number of groupies in the multitype random graph $G(n,K_q)$. Denote by $\alpha =(\alpha _i)\in \mathbb {R}^q$ and $\beta =(\beta _{ij})\in \mathbb {R}^{q\times q}$. For generality, we will usually think of $\beta$ and $\alpha$ as functions of *n* in the same spirit of random graph theory (Bollobás 2001; Janson et al. 2000). Let $\mathbf{1}=(1,\ldots ,1)^T\in \mathbb {R}^q$ be the all-one vector. All the asymptotic notations used in the paper such as *O*, *o*, and $\Omega$ are standard, see e.g. Janson et al. (2000). Our first result is as follows.

### **Theorem 1**

*Let*$q\ge 2$. *Assume that*$\beta \alpha =(\theta +o(\sqrt{\ln n}/n))\mathbf{1}$, *where*$\theta >0$*is a constant. If*$\min _{i\not =j}\{\alpha _i,\beta _{ij}\}>c$*for some constant*$c>0$, *and*$\max _{i}\{\beta _{ii}\}=o(\sqrt{\ln n}/n)$, *then*

$$\begin{aligned} {\text {P}}\left( \frac{\alpha _in}{2}-\omega (n)\sqrt{n}\le N_i\le \frac{\alpha _in}{2}+\omega (n)\sqrt{n},\ for\ i=1,\ldots ,q\right) \rightarrow 1 \end{aligned}$$

*as*$n\rightarrow \infty$, *where*$\omega (n)=\Omega (\ln n)$*is any function tending to infinity. Hence,*

$$\begin{aligned} {\text {P}}\left( \frac{n}{2}-\omega (n)\sqrt{n}\le N\le \frac{n}{2}+\omega (n)\sqrt{n}\right) \rightarrow 1 \end{aligned}$$

*as*$n\rightarrow \infty$, *where*$\omega (n)=\Omega (\ln n)$*is any function tending to infinity.*

When $\beta$ and $\alpha$ are independent of *n*, the following corollary is immediate.

### **Corollary 1**

*Let*$q\ge 2$. *Assume that*$\beta \alpha =\theta \mathbf{1}$*for*$\theta >0$, *and*$\beta _{ii}=0$*for alli*. *Then*

$$\begin{aligned} {\text {P}}\left( \frac{\alpha _in}{2}-\omega (n)\sqrt{n}\le N_i\le \frac{\alpha _in}{2}+\omega (n)\sqrt{n},\ for\ i=1,\ldots ,q\right) \rightarrow 1 \end{aligned}$$

*as*$n\rightarrow \infty$, *where*$\omega (n)=\Omega (\ln n)$*is any function tending to infinity.*

Clearly, by taking $q=2$, $\alpha _1=\alpha _2=1/2$, and $\beta _{11}=\beta _{22}=0$, we recover the result in Shang (2010, Thm. 1) for balanced random bipartite graphs.

Theorem 1 requires that the edges between sets $V_i$, $i=1,\ldots ,q$ are dense, namely, the multitype random graph $G(n,K_q)$ in question resembles a dense ‘multipartite’ graph. For sparse random graphs on the other hand, we have the following result.

### **Theorem 2**

*Let*$q\ge 1$. *Assume that*$\beta \alpha =(\theta +o(\sqrt{\ln n}/n))\mathbf{1}$, *where*$\theta =\theta (n)$*is a function ofn*. *If*$\min _{i}\{\alpha _i\}>c$*for some constant*$c>0$, $\min _{i\not =j}\{\beta _{ij}\}\gg (\ln n)^2/n$, *and*$\max _{i}\{\beta _{ii}\}=o(\sqrt{\ln n}/n)$, *then*

$$\begin{aligned} {\text {P}}\left( \frac{\alpha _in(1-\varepsilon (n))}{2}\le N_i\le \frac{\alpha _in(1+\varepsilon (n))}{2},\ for\ i=1,\ldots ,q\right) \rightarrow 1 \end{aligned}$$

*as*$n\rightarrow \infty$, *where*$\varepsilon (n)=\Omega (\ln n/\sqrt{n})$*is any function tending to zero. Hence,*

$$\begin{aligned} {\text {P}}\left( \frac{n(1-\varepsilon (n))}{2}\le N\le \frac{n(1+\varepsilon (n))}{2}\right) \rightarrow 1 \end{aligned}$$

*as*$n\rightarrow \infty$, *where*$\varepsilon (n)=\Omega (\ln n/\sqrt{n})$*is any function tending to zero.*

It follows from Theorem 2 that we may reproduce the result for sparse Erdős-Rényi random graphs Fernandez de la Vega and Tuza (2009, Thm. 2) by taking $q=\alpha _1=1$, $\beta _{11}=o(\sqrt{\ln n}/n)$; and the result for sparse balanced random bipartite graphs Shang (2010, Thm. 2) by taking $q=2$, $\alpha _1=\alpha _2=1/2$, $\beta _{11}=\beta _{22}=0$ and $\beta _{12}\gg (\ln n)^2/n$.

The multitype random graph $G(n,K_q)$ is generated through a double random process. In the following, we will also consider a closely related ‘random-free’ model $G'(n,K_q)$. Given a gene $K_q$ defined as above, the *random-free multitype random graph*$G'(n,K_q)$ (a.k.a. stochastic block model Holland et al. 1983) is constructed by partitioning [*n*] into *q* sets $V_1,\ldots ,V_q$ with $|V_i|=\alpha _in$. Recall that $\sum _{i=1}^q\alpha _i=1$. We draw an edge *vu* with probability $\beta _{ij}$ independently for $v\in V_i$ and $u\in V_j$; thus the first random step in the original construction disappears, which explains the name ‘random-free’.

In "Proof of the main results" section, we will show Theorems 1 and 2 by first proving analogous results for the random-free version $G'(n,K_q)$. To illustrate our theoretical results, a numerical example is presented in "Numerical simulations" section.

## Proof of the main results

### **Proposition 1**

*Theorem* 1*holds verbatim for the random-free model*$G'(n,K_q)$.

### *Proof*

Without loss of generality, we consider $i=1$, other values of *i* being completely similar. Take vertex $v\in V_1$ and denote by $d_v$ the degree of *v* in $G'(n,K_q)$. Therefore, $d_v=\sum _{i=1}^qd_i$, where $d_i$ means the number of neighbors of *v* in $V_i$. Let $S_v$ represent the sum of degrees of the neighbors of *v*. Write ${\text {Bin}}(n,p)$ for a Binomial variable with parameters *n* and *p*. Assuming that *v* has degree $d_v$, we obtain

1 $$\begin{aligned} S_v\sim&\ d_v+2\sum _{i=1}^q{\text {Bin}}\left( \frac{d_i(d_i-1)}{2},\beta _{ii}\right) +2\sum _{1\le i<j\le q}{\text {Bin}}(d_id_j,\beta _{ij})\nonumber \\&\ +\sum _{j=1}^q{\text {Bin}}\left( d_j(\alpha _1n-d_1-1),\beta _{1j}\right) +\sum _{i=2}^q\sum _{j=1}^q{\text {Bin}}\left( d_j(\alpha _in-d_i),\beta _{ij}\right) , \end{aligned}$$

where the second and third terms on the right-hand side evaluate the contribution of degrees within the neighborhood, while the last two terms correspond to the sum of out-going degrees. Here, $\sim$ means identity of distribution by convention.

For any $d_v$, the expectation of $S_v$ can be computed as

2 $$\begin{aligned} {\text {E}}S_v=d_v+\sum _{i=1}^qd_i^2\beta _{ii}-\sum _{i=1}^qd_i\beta _{ii}-\sum _{j=1}^qd_j\beta _{1j}+\sum _{i=1}^q\sum _{j=1}^qd_j\alpha _in\beta _{ij}. \end{aligned}$$

It follows from the assumption $$\max _{i}\{\beta _{ii}\}=o(\sqrt{\ln n}/n)$$ and the reverse Cauchy–Schwarz inequality Pólya and Szegö (1972, p. 71) that $$\sum _{i=1}^qd_i^2\beta _{ii}=o(d_v^2\sqrt{\ln n}/n)$$. Using $$\beta \alpha =(\theta +o(\sqrt{\ln n}/n))\mathbf{1}$$ and the symmetry of $$\beta $$, we obtain $$\sum _{i=1}^q\sum _{j=1}^qd_j\alpha _in\beta _{ij}=\sum _{j=1}^qd_j\sum _{i=1}^q\alpha _in\beta _{ji}$$$$=d_v(\theta n+o(\sqrt{\ln n}))$$. Consequently, (2) becomes $${\text {E}}S_v=d_v\theta n+\Theta (d_v)+o(d_v^2\sqrt{\ln n}/n)+o(d_v\sqrt{\ln n})$$. Define the event $$\mathcal {A}_v=\{\alpha _1\beta _{11}n-(\ln n)\sqrt{n\beta _{11}}\le d_1\le \alpha _1\beta _{11}n+(\ln n)\sqrt{n\beta _{11}},\ \alpha _i\beta _{1i}n-(\ln n)\sqrt{n}\le d_i\le \alpha _i\beta _{1i}n+(\ln n)\sqrt{n},\ \mathrm {for}\ i=2,\cdots ,q\}$$. Set $$\Phi =\sum _{i=1}^qd_i(d_i-1)/2+\sum _{i<j}d_id_j+\sum _{j=1}^qd_j(\alpha _1n-d_1-1)+\sum _{i=2}^q\sum _{j=1}^qd_j(\alpha _in-d_i)$$. In view of (1), the distribution of $$S_v-d_v$$ is identical to that of the sum of $$\Phi $$ independent random variables, each of which is bounded above by 2. This number is $$\Theta (n^2)$$ when the event $$\mathcal {A}_v$$ occurs. Thus, the large deviation bound Janson et al. (2000, p. 29) gives

$$\begin{aligned} {\text {P}}(|S_v-d_v\theta n|\le 10qn\sqrt{\ln n}\ |\mathcal {A}_v) & \ge{\text {P}}(|S_v-d_v-{\text {E}}(S_v-d_v)|\le 8qn\sqrt{\ln n}\ |\mathcal {A}_v)\\ & \ge 1-e^{-3\ln n}=1-o(n^{-1}). \end{aligned}$$

Dividing by $d_v$ and noting that $q\ge 2$, we obtain for any constant $C_1\ge \frac{11}{c^2}$

$$\begin{aligned} {\text {P}}\Big (\big |\frac{S_v}{d_v}-\theta n\big |\le C_1\sqrt{\ln n}\ \Big |\mathcal {A}_v\Big )=1-o(n^{-1}). \end{aligned}$$

Furthermore, it is straightforward to check that the event $\mathcal {A}_v$ holds with probability $1-o(n^{-1})$ using the Chernoff bound Janson et al. (2000, p. 27) and the fact $d_1\sim {\text {Bin}}(\alpha _1n-1,\beta _{11})$ and $d_i\sim {\text {Bin}}(\alpha _in,\beta _{1i})$, $i=2,\cdots ,q$. Therefore, an application of the total probability formula yields

3 $$\begin{aligned} {\text {P}}\Big (\big |\frac{S_v}{d_v}-\theta n\big |\le C_1\sqrt{\ln n},\ \mathrm {for}\ \mathrm {every}\ v\in V_1\Big )=1-o(1). \end{aligned}$$

Now denote by $N_1^+$ the number of vertices in $V_1$, whose degrees are at least $\theta n+C_1\sqrt{\ln n}$. Similarly, denote by $N_1^-$ the number of vertices in $V_1$, whose degrees are at most $\theta n-C_1\sqrt{\ln n}$. The estimation (3) implies that

$$\begin{aligned} {\text {P}}(N_1^+\le N_1\le \alpha _1n-N_1^-)=1-o(1), \end{aligned}$$

where we recall the definition of $N_1$ as the number of groupies in $V_1$. To complete the proof, it suffices to show

4 $$\begin{aligned} {\text {P}}\Big (N_1^+\ge \frac{\alpha _1n}{2}-\omega (n)\sqrt{n}\Big )=1-o(1) \end{aligned}$$

and the analogous statement for $N_1^-$, where $\omega (n)=\Omega (\ln n)$ is any function tending to infinity.

We write $N_1^+$ as the sum of indicators, namely, $N_1^+=\sum _{v\in V_1}1_{\{d_v\ge \theta n+C_1\sqrt{\ln n}\}}$. Notice that $d_v\sim {\text {Bin}}(\alpha _1n-1,\beta _{11})+\sum _{i=2}^q{\text {Bin}}(\alpha _in,\beta _{1i})$ is a sum of independent binomial variables. Since $d_v$ is flat around its maximum (Butler and Stephens 1993; Drezner and Farnum 2007), we obtain

$$\begin{aligned} {\text {E}}N_1^+=\alpha _1n\cdot {\text {P}}(d_v\ge \theta n+C_1\sqrt{\ln n})=\frac{\alpha _1n}{2}-\Theta (\sqrt{n\ln n}). \end{aligned}$$

Based on the bounded difference inequality (see e.g. Bollobás (2001, p. 24) with the difference $c_k\equiv 1$), we obtain for any $\omega (n)=\Omega (\ln n)$,

$$\begin{aligned} {\text {P}}\Big (N_1^+\le \frac{\alpha _1n}{2}-\omega (n)\sqrt{n}\Big )={\text {P}}(N_1^+\le {\text {E}}N_1^+-\omega '(n)\sqrt{n}) \le e^{-\frac{2\omega '^2(n)n}{n}}=o(1), \end{aligned}$$

where $\omega '(n)$ is a function tending to infinity as $n\rightarrow \infty$. This proves (4). Following the same reasoning we can show ${\text {P}}(N_1^-\ge \alpha _1n/2-\omega (n)\sqrt{n})=o(1)$, which concludes the proof. $\square$

### **Proposition 2**

*Theorem* 2*holds verbatim for the random-free model*$G'(n,K_q)$, *except that we herein allow*$\varepsilon (n)=\Omega (\sqrt{\ln n/n})$*as any function tending to zero.*

### *Proof*

We sketch the proof as it is similar. As in the proof of Proposition 1, we consider $i=1$ and obtain the expectation of $S_v$ for $v\in V_1$ as ${\text {E}}S=d_v\theta n+\Theta (d_v)+o(d_v^2\sqrt{\ln n}/n)+o(d_v\sqrt{\ln n})$. Define the event $\mathcal {B}_v=\{\alpha _i\beta _{1i}n-(\ln n)\sqrt{n\beta _{1i}}\le d_i\le \alpha _i\beta _{1i}n+(\ln n)\sqrt{n\beta _{1i}},\ \mathrm {for}\ i=1,\ldots ,q\}$. The Chernoff bound Janson et al. (2000, p. 27) implies that $\mathcal {B}_v$ holds with probability $1-o(n^{-1})$. Using the large deviation bound Janson et al. (2000, p. 29) we obtain

$$\begin{aligned} {\text {P}}\big (|S_v-d_v\theta n|\le 10q\ln ^{\frac{5}{2}}n\ \big |\mathcal {B}_v\big )& \ge {\text {P}}\big (|S_v-d_v-{\text {E}}(S_v-d_v)|\le 8q\ln ^{\frac{5}{2}} n\ \big |\mathcal {B}_v\big )\\ & \ge 1-e^{-2\ln n}=1-o(n^{-1}). \end{aligned}$$

Dividing by $d_v$, we obtain similarly for some constant $C_2>0$

$$\begin{aligned} {\text {P}}\Big (\big |\frac{S_v}{d_v}-\theta n\big |\le C_2\sqrt{\ln n}\ \Big |\mathcal {B}_v\Big )=1-o(n^{-1}), \end{aligned}$$

and

5 $$\begin{aligned} {\text {P}}\Big (\big |\frac{S_v}{d_v}-\theta n\big |\le C_2\sqrt{\ln n},\ \mathrm {for}\ \mathrm {every}\ v\in V_1\Big )=1-o(1). \end{aligned}$$

Denote by $N_1^+$ the number of vertices in $V_1$, whose degrees are at least $\theta n+C_2\sqrt{\ln n}$. Denote by $N_1^-$ the number of vertices in $V_1$, whose degrees are at most $\theta n-C_2\sqrt{\ln n}$. The result (5) again implies that

$$\begin{aligned} {\text {P}}(N_1^+\le N_1\le \alpha _1n-N_1^-)=1-o(1). \end{aligned}$$

It remains to show

6 $$\begin{aligned} {\text {P}}\bigg (N_1^+\ge \frac{\alpha _1n(1-\varepsilon (n))}{2}\bigg )=1-o(1) \end{aligned}$$

and the analogous statement for $N_1^-$, where $\varepsilon (n)=\Omega (\sqrt{\ln n/n})$ is any function tending to zero.

Set $N_1^+=\sum _{v\in V_1}1_{\{d_v\ge \theta n+C_2\sqrt{\ln n}\}}$. As in the proof of Proposition 1, we arrive at

$$\begin{aligned} {\text {E}}N_1^+=\alpha _1n\cdot {\text {P}}(d_v\ge \theta n+C_2\sqrt{\ln n})=\frac{\alpha _1n}{2}-\Theta (\sqrt{n\ln n}). \end{aligned}$$

Invoking the bounded difference inequality Bollobás (2001, p. 24) and taking $\omega (n):=\varepsilon (n)\sqrt{n}\rightarrow \infty$, we obtain for any $\varepsilon (n)=\Omega (\sqrt{\ln n/n})$,

$$\begin{aligned} {\text {P}}\bigg (N_1^+\le \frac{\alpha _1n(1-\varepsilon (n))}{2}\bigg )={\text {P}}(N_1^+\le {\text {E}}N_1^+-\omega (n)\sqrt{n}) \le e^{-2\omega ^2(n)}=o(1), \end{aligned}$$

as $n\rightarrow \infty$. This completes the proof of (6). Likewise, we have ${\text {P}}(N_1^-\ge \alpha _1n(1-\varepsilon (n))/2)=o(1)$ as desired. $\square$

### *Proof of Theorem 1 and Theorem 2*

These results can be proven in the similar way as Propositions 1 and 2 by noting that, in the $G(n,K_q)$ model, ${\text {P}}(||V_i|-\alpha _in|\ge \ln n\sqrt{n})=o(n^{-1})$ for all $i=1,\ldots ,q$. $\square$

## Numerical simulations

To illustrate our theoretical results, in this section we present a numerical example for the $G(n,K_q)$ model with $q=3$.

**Fig. 1:**
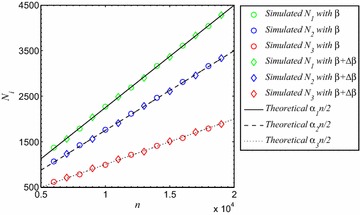
Number of groupies $N_i$ $(i=1,2,3)$ versus number of vertices in $G(n,K_3)$ with $\alpha _1=0.45$, $\alpha _2=0.35,$ $\alpha _3=0.2$, and two different choices of $\{\beta _{ij}\}$. Each data point is obtained by averaging over a sample of 50 independent random graphs

Set $\alpha =(0.45,0.35,0.2)^T$, $$\beta =\left( \begin{array}{ccc}0&{} 8/21&{}1/3\\ 8/21&{}0&{}1/7\\ 1/3&{}1/7&{}0\end{array}\right) $$, and $\theta =0.2$. In Fig. 1 we plot the numbers of groupies $N_i$ for $i=1,2,3$ as functions of *n*, (i) with the above constant $\beta$; and (ii) with perturbed $\beta +\Delta \beta$, where $\Delta \beta =(\ln ^{1/4}n)/n\mathbf{1}\mathbf{1}^T$. Clearly, the conditions in Theorem 1 hold for both situations (i) and (ii). Fig. 1 shows that the agreement between the simulations and the theoretical prediction of Theorem 1 is excellent.

## Conclusion

In this paper, we have studied the groupies in multitype random graphs. It is discovered that the proportion of groupies is very close to 1/2 in multitype random graphs, which include Erdős-Rényi random graphs, random bipartite, and multipartite graphs as special examples. We mention that there are several possibilities to continue this line of research, both by considering other more realistic random network models as well as by analyzing the limit distribution of groupies in random graphs. For example, a natural question could be to ask if there are similar results for $q=q(n)$ or edge-independent random graphs (e.g. Shang 2016)?
